# ﻿Three new species of Valvatidae, Planorbidae, and Lymnaeidae (Mollusca, Gastropoda) from Lake Bitahai, Yunnan Plateau, China

**DOI:** 10.3897/zookeys.1264.170832

**Published:** 2025-12-19

**Authors:** Le-Jia Zhang, Shu-Wei Liu, Xiao-Yong Chen

**Affiliations:** 1 Yunnan Key Laboratory of Biodiversity and Ecological Conservation of Gaoligong Mountain, Kunming Institute of Zoology, Chinese Academy of Sciences, Kunming 650023, China Kunming Institute of Zoology, Chinese Academy of Sciences Kunming China; 2 Southeast Asia Biodiversity Research Institute, Chinese Academy of Sciences, Yezin, Nay Pyi Taw, 05282, Myanmar Southeast Asia Biodiversity Research Institute Nay Pyi Taw Myanmar; 3 Yunnan International Joint Laboratory of Southeast Asia Biodiversity Conservation, Mengla 666303, China Yunnan International Joint Laboratory of Southeast Asia Biodiversity Conservation Mengla China

**Keywords:** Endemic species, freshwater snail, molecular phylogeny, Potatso National Park, radicine pond snails, taxonomy

## Abstract

Three new species of freshwater snails are described from Lake Bitahai, a small plateau lake located in the high altitude region of northwestern Yunnan, China: *Valvata bitaensis* Zhang, **sp. nov.**, *Gyraulus semskyinyizla* Zhang, **sp. nov.**, and *Radix shangrila* Zhang, **sp. nov.** These new species can be distinguished from their congeners by morphology and molecular phylogenetic analyses. *Valvata bitaensis* Zhang, **sp. nov.** has an aperture that is always detached from the body whorl, while its shell is sometimes uncoiled. *Gyraulus semskyinyizla* Zhang, **sp. nov.** can be distinguished from its sister species *Gyraulus chinensis* by its rough periostracum and mantle roof with dense fine black dots. *Radix shangrila* Zhang, **sp. nov.** can be distinguished from its congeners by the morphology of its columellar lip and mantle pigments. Following the discovery of these three new freshwater snail species, Lake Bitahai now harbours five endemic species, highlighting its significant value for biodiversity conservation.

## ﻿Introduction

Lake Bitahai is a plateau lake located in Potatso National Park of Shangri-La City in northwestern Yunnan, China. This lake lies at the intersection of Qinghai-Xizang Plateau and the Hengduan Mountains biodiversity hotspot, covering an area of about 1.4 km^2^, at an elevation of 3539 m, with a maximum depth of around 40 m (Wang and Dou 1998). The Potatso National Park harbours at least 280 vertebrate species and 493 species of insects, and the wetland surrounding Lake Bitahai represents one of the most important wintering sites of the Black-necked Crane and many other migratory birds in the region (Zhou and Chen 2006). One fish species (*Ptychobarbus chungtienensis* Tsao, 1964) and one amphipod species (*Gammarus bitaensis* Shu, Yang & Chen, 2012) are endemic to the lake (Zhou and Chen 2006; Shu et al. 2012).

Many plateau lakes in Yunnan exhibit high biodiversity and endemism, especially in freshwater molluscs. A large number of new species, genera or even families of freshwater molluscs have been reported from these plateau lakes in recent years (e.g., Zhang et al. 2015; Zhang 2017; Zhang et al. 2023; Fan et al. 2024; Xiang et al. 2025). The only published paper involving freshwater molluscs from Lake Bitahai is that of Du et al. (2012), who investigated the diversity of freshwater molluscs in four plateau lakes of northwestern Yunnan; altogether six species have been recorded from Lake Bitahai: *Radix yunnanensis* Nevill, 1877, *Radix swinhoei* (Adams, 1866), *Physa acuta* Draparnaud, 1805 (= *Physella acuta*), *Valvata* sp., *Pisidium* sp., and *Sphaerium* sp. There is no taxonomic study of freshwater molluscs from Lake Bitahai using morphological and molecular methods.

In July 2025, we conducted fieldwork at Lake Bitahai and collected freshwater molluscs. Among these specimens, three species were morphologically different from known taxa. We here formally describe these species as new to science, based on a comprehensive study of their morphology and molecular phylogeny.

## ﻿Material and methods

### ﻿Materials

**Figure 1.**
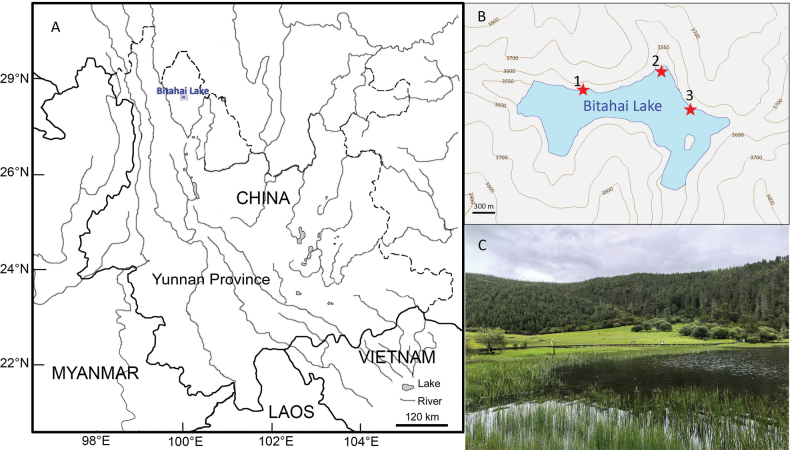
Collecting sites of freshwater snails in Lake Bitahai. A. Map of Yunnan Province showing the location of Lake Bitahai, modified from Zhang et al. (2015); B. Topographic map showing three collecting sites (red stars) in Lake Bitahai; C. Habitat at site 2.

Specimens of freshwater snails were collected by hand from Lake Bitahai, mostly at three sites (Fig. 1B). Specimens were kept in ethanol, and deposited in the collection of the Kunming Institute of Zoology, Chinese Academy of Sciences, Kunming (**KIZ**). Ten ethanol-preserved specimens kept in KIZ were used for PCR amplification.

### ﻿Examination of morphology

The height (H) and width (W) of shells of mature and complete specimens were measured with an electronic calliper to a precision of 0.1 mm. The specimens were photographed with a Nikon Z5 camera. The morphological and anatomical descriptions of the studied species were carried out mainly by analogy with the articles of Nevill (1877), Zhang et al. (1997), Beran and Glöer (2006), Shu et al. (2013), Aksenova et al. 2019, Vinarski et al. (2020) and Fan et al. (2024).

### ﻿DNA extraction and sequencing

DNA was extracted from 10–20 mg of foot tissue from each snail using Vazyme FastPure Cell/Tissue DNA Isolation Mini Kit-box 1. A fragment of the mitochondrial cytochrome *c* oxidase subunit I (COI) gene was amplified through polymerase chain reaction (PCR) with the primer pair LCO1490 and COX-B7R (Schultheiß et al. 2011) PCR amplifications were conducted in 30 μl volumes under the following cycling conditions: initial denaturing step at 94 °C for 10 min, followed by 30 cycles of 94 °C for 1 min, 50 °C for 1 min, and 72 °C for 1 min, with a final extension step of 10 min at 72 °C. The purification and sequencing were conducted by Sangon Biotech, Shanghai, China.

### ﻿Sequence and phylogenetic analyses

Ten new COI sequences (two Valvatidae, four Planorbidae, and four Lymnaeidae) of the three species from Lake Bitahai and altogether 31 sequences of Valvatidae, 42 sequences of Planorbidae and 56 sequences of Lymnaeidae, mostly from von Oheimb et al. (2011), von Oheimb et al. (2013), Clewing et al. (2015), Wiese et al. (2020), Saito et al. (2021), Fan et al. (2024), and Chen et al. (2025), were included in the present study (Suppl. materials 1–3, alignment of COI sequences of Valvatidae, alignment of COI sequences of Planorbidae, alignment of COI sequences of Lymnaeidae). *Cornirostra pellucida* (GenBank ID: AY296842) was chosen as the outgroup of the Valvatidae tree according to Clewing et al. (2014); *Planorbis planorbis* (GenBank ID: MZ127287) was chosen as the outgroup of the Planorbidae tree according to Chen et al. (2025); *Ampullaceana fontinalis* (GenBank ID: EU818802) was chosen as the outgroup of the *Radix* tree according to Vinarski et al. (2020).

Sequences were aligned using MUSCLE as implemented in Geneious Prime 2020 (https://www.geneious.com). Uncorrected p-distances were calculated using Geneious Prime 2020. The best-fit model of sequence evolution for the dataset was selected using the Akaike information criterion in MEGA X (Kumar et al. 2018). A Bayesian inference (BI) analysis was performed with MrBayes v. 3.2.6 (Ronquist et al. 2012) as implemented in Geneious Prime 2020 with four independent chains for 20000000 generations, sample freq = 4000, burnin = 25%, and we confirmed that convergence was reached based on the trace plots generated in Geneious Prime 2020. Nodal support was assessed by estimating posterior probabilities (pp).

## ﻿Results

### ﻿Family Valvatidae Gray, 1840

**Genus *Valvata* O.F. Müller, 1773**

#### ﻿ Valvata bitaensis

Taxon classificationAnimaliaMolluscaValvatidae

Zhang sp. nov.

415560C2-8449-5168-A127-BEF835B4DFCA

https://zoobank.org/06502ED4-80F9-4BCA-B7BB-86A1DE016A74

##### Material examined.

***Holotype***: KIZ. 2500001, site 2 in Lake Bitahai, Shangri-La City, Yunnan Province, China, on submerged wood, 3550 m above sea level. ***Paratypes***: 8 specimens, KIZ. 2500002–2500009, 2500002 referring to VB2 and 2500009 referring to VB1 in COI tree, site 1 and 2 in Lake Bitahai, Shangri-La City, Yunnan Province, China, on submerged wood and rocks, around 3540 m above sea level.

##### Etymology.

Named after the type locality Lake Bitahai. The recommended Chinese name is “碧塔盘螺”.

##### Description.

**Figure 2.**
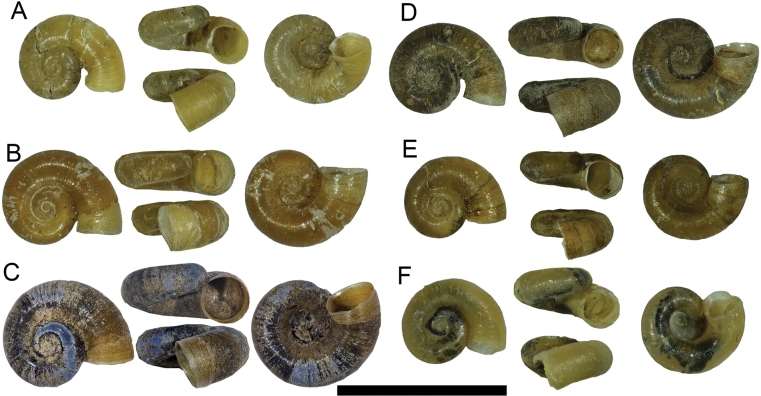
Shell of *Valvata bitaensis* sp. nov. A. Holotype; B–F. Paratypes; F. VB2 in BI tree of Fig. 4. Scale bars: 5 mm.

**Figure 3.**
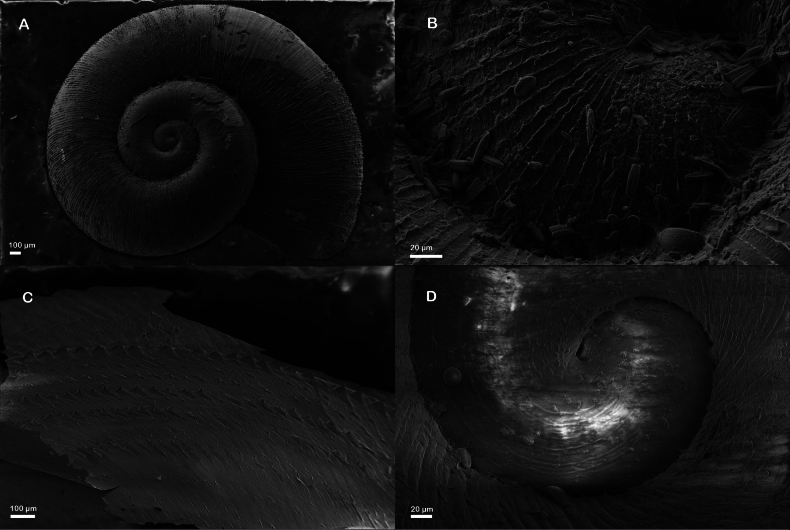
SEM photo of freshwater snails from Lake Bitahai. A. Adult shell of *Valvata bitaensis* sp. nov.; B. Surface structure of embryonic shell of *Valvata bitaensis* sp. nov.; C. Adult shell surface structure of *Gyraulus semskyinyizla* sp. nov.; D. Embryonic shell of *Gyraulus semskyinyizla* sp. nov.

Shell very small (Table 1), light yellow to brown, thin, discoidal, with 3–3.5 regularly increasing whorls; shell surface with dense fine growth lines both in adult and embryonic shell, spiral whorls flat disc-shaped, last 1/5 body whorl always uncoiling and twisting diagonally downward; aperture round, inner lip always detached from body whorl, umbilicus widely open, nearly all whorls visible from umbilicus side. Operculum round, thin, concave, 6–7 dextral whorls (Figs 2, 3A, B).

**Table 1.** Shell measurements (in mm) of the three new species of freshwater snail from Lake Bitahai. The median and standard deviation are provided for shell height (H), width (W), and the width-to-height ratio (W/H).

Species	*N*	Height (H)	Width (W)	W/H
*Valvata bitaensis* sp. nov.	9	1.7±0.3	3.3±0.3	1.99±0.29
*Gyraulus semskyinyizla* sp. nov.	10	1.8±0.1	5.7±0.4	3.25±0.19
*Radix shangrila* sp. nov.	9	16.9±2.0	10.1±1.3	0.51±0.08

##### Habitat and distribution.

Only known from Lake Bitahai. Prefers hard substrates such as rocks and submerged wood.

##### COI sequence data.

**Figure 4.**
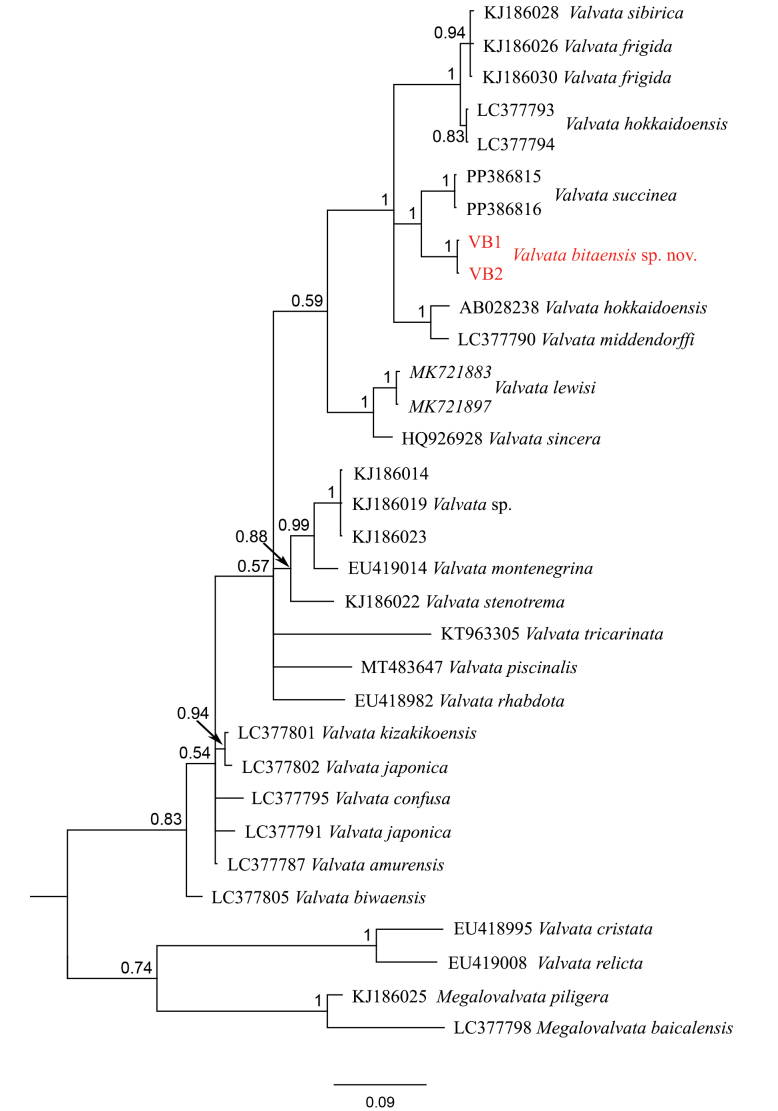
BI tree of partial COI sequences of Valvatidae. *Valvata bitaensis* sp. nov. is marked in red.

The COI phylogeny (Fig. 4) shows that *Valvata bitaensis* sp. nov. forms a well-supported sister clade (pp = 1) of *Valvata succinea* endemic to Lake Lugu of northwestern Yunnan (pp = 1). The uncorrected p-distance of COI between these two species is around 4.5%.

##### Remarks.

This species has fine, dense ribs (Fig. 3A, B) and the inner lip of the aperture is always detached from the body whorl. Sometimes the shell is uncoiled, by which it can be easily distinguished from all known *Valvata* species, especially *Valvata succinea* Chen, He & Fan, 2024 another endemic *Valvata* species of Yunnan’s lakes.

### ﻿Family Planorbidae Rafinesque, 1815

**Genus *Gyraulus* Charpentier, 1837**

#### ﻿ Gyraulus semskyinyizla

Taxon classificationAnimaliaMolluscaPlanorbidae

Zhang sp. nov.

AC094073-76E1-5794-8522-01ABCBDDAE3B

https://zoobank.org/3908E8D7-BBD0-4B04-9A2B-368F73FBFBD0

##### Material examined.

***Holotype***: KIZ. 2500010, referring to GS1 in COI tree, site 1 in Lake Bitahai, Shangri-La City, Yunnan Province, China, on submerged rock near shore, 3550 m above sea level. ***Paratypes***: 9 specimens, KIZ. 2500011–2500019, 2500011 referring to GS2, 2500012 referring to GS3, and 2500013 referring to GS4 in COI tree, sites 1, 2 and 3 in Lake Bitahai, Shangri-La City, Yunnan Province, China, on submerged wood and rocks, around 3540 m above sea level.

##### Etymology.

Named after the Tibetan name of Shangri-La City,” སེམས་ཀྱི་ཉི་ཟླ་གྲོང་ཁྱེར།” (Sems kyi nyi zla). The recommended Chinese name is “日月旋螺”.

##### Description.

**Figure 5.**
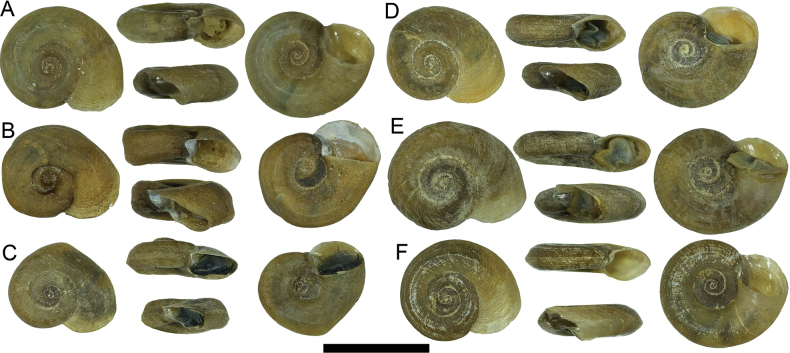
Shell of *Gyraulus semskyinyizla* sp. nov. A. Holotype, GS1 in BI tree of Fig. 7; B–F. Paratypes; B. GS3 in BI tree of Fig. 7; C. GS4 in BI tree of Fig. 7; F. GS2 in BI tree of Fig. 7. Scale bars: 5 mm.

**Figure 6.**
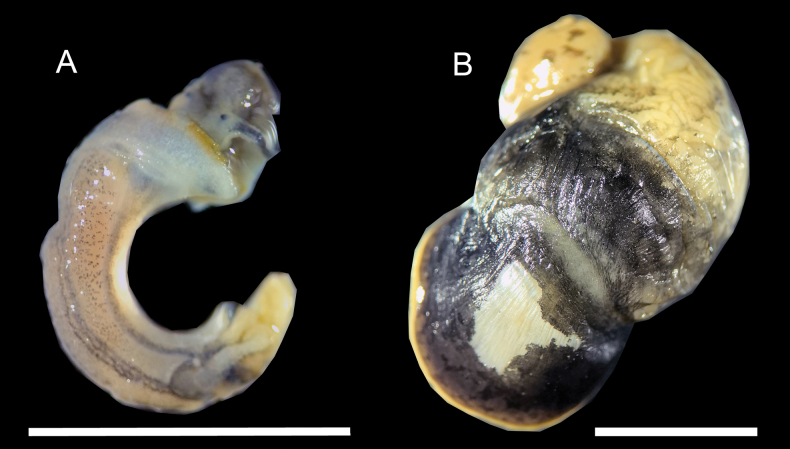
Soft body of *Gyraulus semskyinyizla* sp. nov. (A) and *Radix shangrila* sp. nov. (B), displaying patterns on mantle roof. Scale bars: 5 mm.

Shell very small (Table 1), Pseudodextral, light yellow, thin, discoidal, with 3.5–4 regularly increasing whorls; shell surface rough, with dense spiral striae and vertical growth lines, forming fine reticulate pattern, with short hairs which follow the spiral striae, several weak keels on each whorl; aperture ovate, inner lip tightly attaching to body whorl, Pseudo-umbilicus widely open, shallow, nearly all whorls visible from pseudo-umbilicus side (Figs 3C, D, 5). Animals light grey, mantle roof with dense fine black dots pattern but normally not visible through the shell (Fig. 6A).

##### Habitat and distribution.

Only known from the locality Lake Bitahai. Prefers hard substrates such as rocks and submerged wood.

##### COI sequence data.

**Figure 7.**
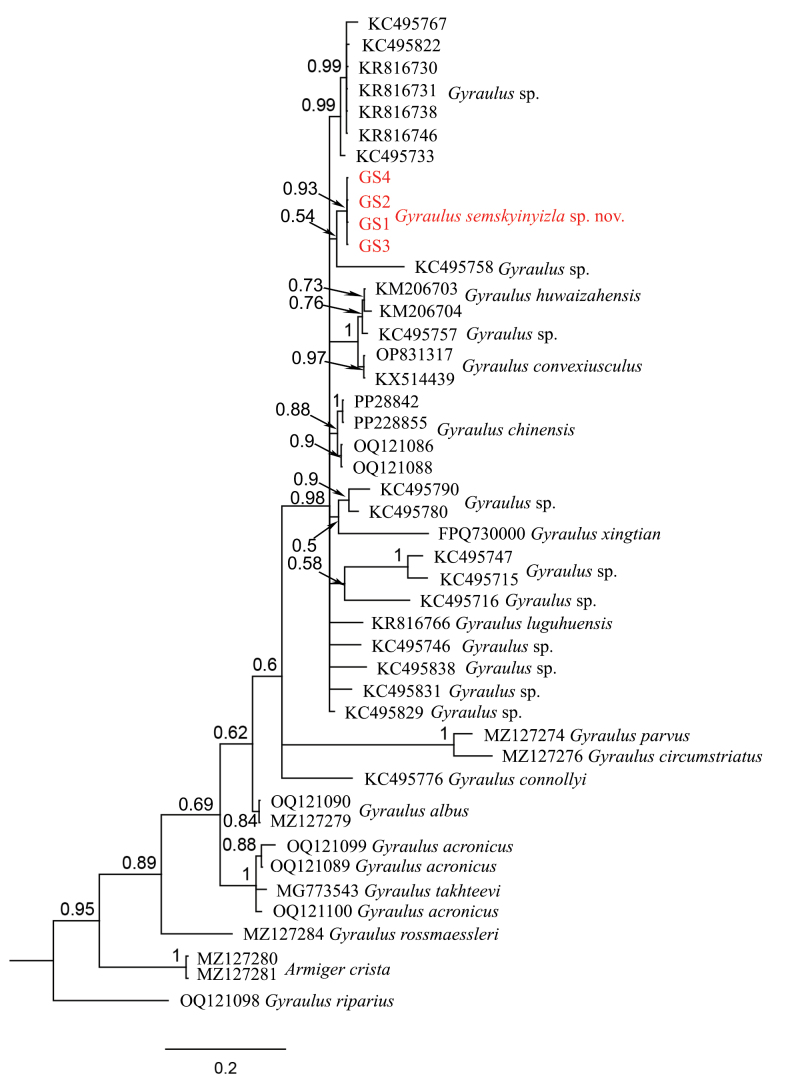
BI tree of partial COI sequences of Planorbidae. *Gyraulus semskyinyizla* sp. nov. is marked in red.

The COI phylogeny (Fig. 7) shows that *Gyraulus semskyinyizla* sp. nov. forms a moderately well-supported clade (pp = 0.93) and falls within a highly supported pectinate clade (pp=0.98) including species from China (*G. chinensis*, *G. xingtian*, *G. luguensis*, *Gyraulus* spp. from Yunnan and Xizang), Nepal (*Gyraulus* spp.), India (*Gyraulus convexiusculus*) and Iraq (*Gyraulus huwaizahensis*). The shortest uncorrected p-distance of COI between *Gyraulus semskyinyizla* sp. nov. and other species within this clade (KC495829*Gyraulus* sp. from Lake Yangzonghai of Yunnan) is around 3.8%

##### Remarks.

This species has a highly variable shell outline, from round (Fig. 5D), keeled (Fig. 5F) to even square-shaped (Fig. 5B). However, it can be distinguished from its congeners, especially *Gyraulus chinensis* (Dunker, 1848), by its (1) rough shell surface with many obvious spiral lines, and (2) mantle roof with dense fine black dots, that are normally not clearly visible through the shell (Fig. 6A). *Gyraulus chinensis* has a relatively smooth shell and a sparse large dots pattern on the mantle that is easily visible through the shell.

### ﻿Family Lymnaeidae Rafinesque, 1815

**Subfamily Amphipepleinae Pini, 1877**

**Tribe Radicini Vinarski, 2013**

**Genus *Radix* Montfort, 1810**

#### ﻿ Radix shangrila

Taxon classificationAnimaliaMolluscaLymnaeidae

Zhang sp. nov.

30B7A3F0-99D4-5FCA-A1CC-0F95F6872B55

https://zoobank.org/F0551A29-F022-4FF4-BCE2-3439CD773324

##### Material examined.

***Holotype***: KIZ. 2500020, referring to RS1 in COI tree, site 1 in Lake Bitahai, Shangri-La City, Yunnan Province, China, on submerged rock near shore, around 3540 m above sea level. ***Paratypes***: 8 specimens, KIZ. 2500021–2500028, 2500021 referring to RS2, 2500022 referring to RS3, and 2500023 referring to RS4 in COI tree, sites 1, 2 and 3 in Lake Bitahai, Shangri-La City, Yunnan Province, China, on submerged wood and rocks, 3550 m above sea level.

##### Etymology.

Named after Shangri-La City. The recommended Chinese name is “中甸萝卜螺”.

##### Description.

**Figure 8.**
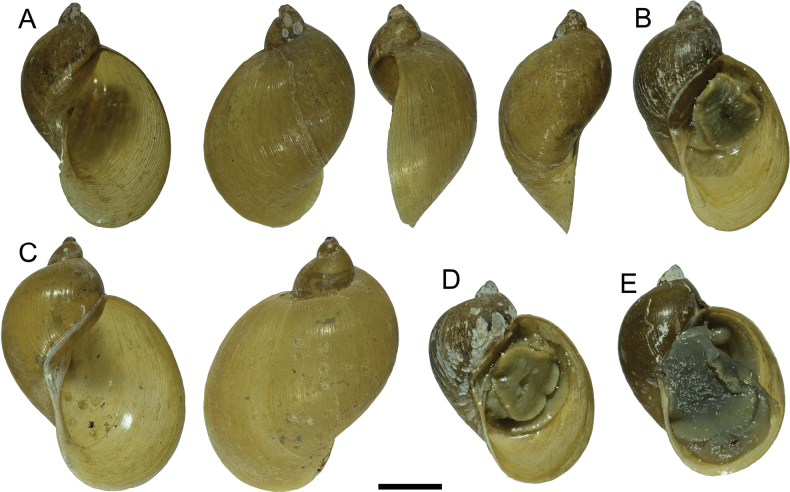
Shell of *Radix shangrila* sp. nov. A. Holotype, RS1 in BI tree of Fig. 9; B–E. Paratypes; B. RS2 in BI tree of Fig. 9; C. RS3 in BI tree of Fig. 9; D. RS4 in BI tree of Fig. 9. Scale bars: 5 mm.

Shell medium (Table 1), yellow, glossy, with 4 whorls enlarging rapidly, spire short and pointed; aperture very large and ear-shaped, columellar fold weak, the lower half of the inner apertural lip straight and sloping outward away from central axis (Fig. 8). Animals light grey, mantle roof with evenly dispersed black-grey pigment, without round spots (Fig. 6B).

##### Habitat and distribution.

Only known from the locality Lake Bitahai. Prefers hard substrates such as rocks and submerged wood.

##### COI sequence data.

**Figure 9.**
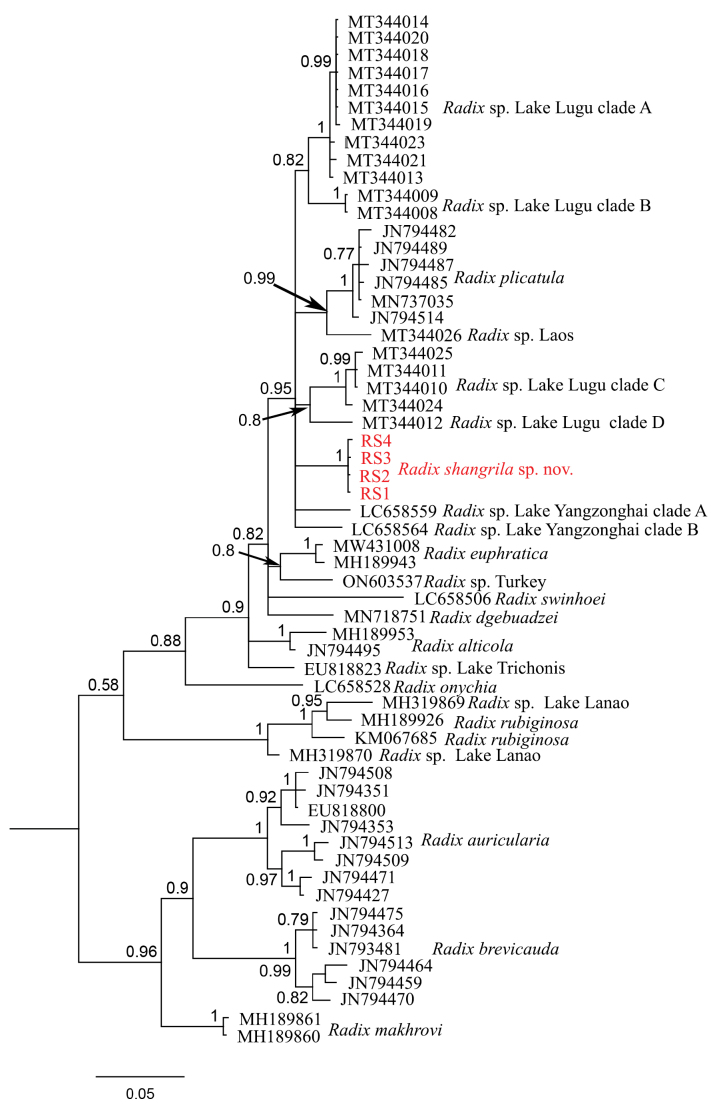
BI tree of partial COI sequences of *Radix* species. *Radix shangrila* sp. nov. is marked in red.

The COI sequence phylogeny (Fig. 9) shows that *Radix shangrila* sp. nov. forms a well-supported clade (pp = 1) within an unresolved polytomy (hexatomy) that includes the Yunnan lakes’ species flock (*Radix* sp. Lake Lugu and *Radix* sp. Lake Yangzonghai), *Radix plicatula*, and *Radix* sp. from Laos (pp = 0.95). The different *Radix* snail morphotypes (Fig. 8A-RS1 in Fig. 9; Fig. 8D-RS4 in Fig. 9) from Lake Bitahai are all *R. shangrila* sp. nov. based on the COI sequence analysis. The shortest uncorrected p-distance of COI between *R. shangrila* sp. nov. and other species within this clade (MT344013*Radix* sp. Lake Lugu clade A) is around 5.1%.

##### Remarks.

Although this species has a variable shell outline (such as Fig. 8A & D), it can be distinguished from its congeners, especially *Radix auricularia* (Linnaeus, 1758), *Radix yunnanensis* Nevill, 1877, and *Radix plicatula* (Benson, 1842), by its (1) weak columellar fold, (2) straight columellar lip sloping outward away from the central axis, and (3) mantle roof without round spots (Fig. 6B). The three other *Radix* species mentioned above, have a relatively strong columellar fold, a curved columellar lip sloping inward to central axis, and a mantle roof with large round spots (Nevill 1877; Vinarski et al. 2020). *Radix yunnanensis* is distributed in Yingjiang County, a tropical region of southwestern Yunnan (Nevill 1877), far from the alpine region of Lake Bitahai in northwestern Yunnan.

## ﻿Discussion

We described three new species of freshwater snails from Lake Bitahai, Yunnan, China. The three new species can be clearly distinguished from their congeners based on morphology and COI sequence analyses. Currently, these three species are only known from Lake Bitahai, bringing the total number of endemic species (including fish and amphipods) in this lake to five. Although Lake Bitahai covers an area of only 1.4 km^2^, it harbours an unexpectedly high number of endemic species, highlighting its significant value for biodiversity conservation.

*Valvata bitaensis* sp. nov. sometimes has an uncoiled shell. Uncoiled shells in *Valvata* were previously recorded in *Valvata lewisi* Currier, 1868 as a rare intraspecific abnormal form, referred to as morph *ontariensis* (Baker 1931; Hinchliffe et al. 2019). Apart from *V. bitaensis* sp. nov., only two other *Valvata* species have been reported in southwestern and western China, viz. *Valvata succinea* from Lake Lugu in northwestern Yunnan (Fan et al. 2024) and *Valvata* sp. from Lake Bangong Co in Xizang (Clewing et al. 2014). The actual diversity of Valvatidae is still considered underestimated in this region.

Two species of Planorbidae were described from Yunnan, China: *Gyraulus luguhuensis* Shu, Köhler, Fu & Wang, 2013 endemic to Lake Lugu, and *Gyraulus xingtian* Chen, He, Xiang & Wu, 2025 endemic to Lake Dianchi (Shu et al. 2013; Chen et al. 2025). Each of these species is restricted to a single lake on the Yunnan Plateau. Similarly, *Gyraulus semskyinyizla* sp. nov. is common in Lake Bitahai but was not found in nearby Lake Napahai. More undescribed *Gyraulus* species are expected to be discovered in other ancient lakes of the Yunnan Plateau.

The diversity of Lymnaeidae on the Yunnan Plateau, a potential hotspot for radicine pond snails, has not been fully explored. Wiese et al. (2020) reported four undescribed lineages of *Radix* species endemic to Lake Lugu, three of which have a unique neritid-like shell shape. Saito et al. (2021) discovered two lineages of *Radix* species (Radix cf. plicatula) endemic to Lake Yangzonghai. The shell outline of *Radix shangrila* sp. nov. is highly variable, similar to that of several other species such as *Radix swinhoei* (Adams, 1866) or *Radix auricularia* (Linnaeus, 1758). However, the morphology of the columellar fold, columellar lip and mantle roof and COI phylogeny strongly support the presence of only a single *Radix* species in Lake Bitahai. Therefore, *Radix yunnanensis* Nevill, 1877 and *Radix swinhoei* (Adams, 1866) from Lake Bitahai reported by Du et al. (2012) are considered misidentifications of *Radix shangrila* sp. nov. Further studies incorporating additional nuclear markers are necessary to reconstruct a robust phylogeny of radicine pond snails from Yunnan.

## Supplementary Material

XML Treatment for Valvata bitaensis

XML Treatment for Gyraulus semskyinyizla

XML Treatment for Radix shangrila

## ﻿Additional information

### Use of AI

No use of AI was reported.

### Author ORCIDs

Le-Jia Zhang https://orcid.org/0000-0002-6002-873X

Shu-Wei Liu https://orcid.org/0009-0006-1010-7938

Xiao-Yong Chen https://orcid.org/0000-0002-0924-5560

### Data availability

All of the data that support the findings of this study are available in the main text or Supplementary Information.

## Supplementary materials

Supplementary material 1 Alignment of COI sequences of Valvatidae Data type fasta https://binary.pensoft.net/file/1496358This dataset is made available under the Open Database License (http://opendatacommons.org/licenses/odbl/1.0/). The Open Database License (ODbL) is a license agreement intended to allow users to freely share, modify, and use this Dataset while maintaining this same freedom for others, provided that the original source and author(s) are credited.Le-Jia Zhang, Shu-Wei Liu, Xiao-Yong Chen

Supplementary material 2 Alignment of COI sequences of Planorbidae Data type fasta https://binary.pensoft.net/file/1496359This dataset is made available under the Open Database License (http://opendatacommons.org/licenses/odbl/1.0/). The Open Database License (ODbL) is a license agreement intended to allow users to freely share, modify, and use this Dataset while maintaining this same freedom for others, provided that the original source and author(s) are credited.Le-Jia Zhang, Shu-Wei Liu, Xiao-Yong Chen

Supplementary material 3 Alignment of COI sequences of Lymnaeidae Data type fasta https://binary.pensoft.net/file/1496360This dataset is made available under the Open Database License (http://opendatacommons.org/licenses/odbl/1.0/). The Open Database License (ODbL) is a license agreement intended to allow users to freely share, modify, and use this Dataset while maintaining this same freedom for others, provided that the original source and author(s) are credited.Le-Jia Zhang, Shu-Wei Liu, Xiao-Yong Chen
